# GWS—A Collaborative Load-Balancing Algorithm for Internet-of-Things

**DOI:** 10.3390/s18082479

**Published:** 2018-07-31

**Authors:** Hongyu Xiao, Zhenjiang Zhang, Zhangbing Zhou

**Affiliations:** 1 Department of Electronic and Information Engineering, Beijing Jiaotong University, Beijing 100044, China; yuhongxiao@aliyun.com; 2 Key Laboratory of Communication and Information Systems, Beijing Municipal Commission of Education, Beijing Jiaotong University, Beijing 100044, China; 3 Department of Software Engineering, Beijing Jiaotong University, Beijing 100044, China; 4 School of Information Engineering, China University of Geosciences at Beijing, Beijing 100083, China; zhangbing.zhou@gmail.com or zbzhou@cugb.edu.cn

**Keywords:** collaborative, Internet-of-Things, fog computing, Nash bargaining solution, Pareto optimality, scheduling, time-sharing

## Abstract

This paper firstly replaces the first-come-first-service (FCFS) mechanism with the time-sharing (TS) mechanism in fog computing nodes (FCNs). Then a collaborative load-balancing algorithm for the TS mechanism is proposed for FCNs. The algorithm is a variant of a work-stealing scheduling algorithm, and is based on the Nash bargaining solution (NBS) for a cooperative game between FCNs. Pareto optimality is achieved through the collaborative working of FCNs to improve the performance of every FCN. Lastly the simulation results demonstrate that the game-theory based work-stealing algorithm (GWS) outperforms the classical work-stealing algorithm (CWS).

## 1. Introduction

**Figure 1:**
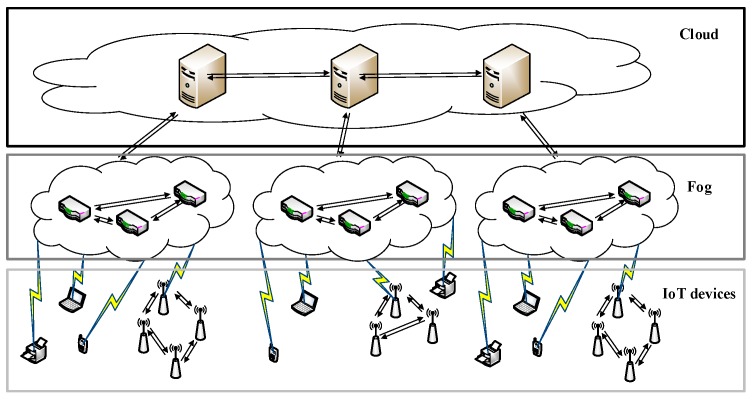
IoT architecture for fog computing.

Along with the rapid development of IoT, fog computing has emerged as a promising architecture for IoT applications. As the necessary complement to cloud computing, fog computing serves IoT devices by undertaking part of their work load. IoT devices typically feature weak computing capacity and low energy. With the help of fog computing, IoT devices can deliver some tasks to a fog computing nodes (FCNs) to relieve their load and reduce the energy consumption [1]. The IoT architecture is shown in Figure 1.

The IoT architecture is planar, comprised of a cloud layer, a fog layer and a layer of IoT devices. The fog layer—the-so-called “fog computing network” helps the cloud to process IoT tasks. Because the fog layer is much closer to the edge, it achieves low latency and quick response time, both of which are necessary for IoT applications. For example, in a wireless sensor network, sensors may utilize the fog computing nodes to do some computing and make decisions. At this point, FCNs simply act as rule engines, so each FCN must respond as quickly as possible for higher quality of service (QoS). If the FCNs respond slowly, then some operations of the wireless sensor network will be delayed.

Fog computing nodes (FCNs), as the key components of fog computing, are always located at the edge in contrast to the cloud computing data centers at the center of the Internet. FCNs have low latency for IoT applications. ‘Latency’, here, refers to the FCN’s response time for IoT tasks. The work mechanism is somewhat important factor influencing latency. The work mechanism of FCNs can be divided into three categories: concurrent, priority and FCFS [2]. When an FCN possesses only one single-core processor, the concurrent mechanism degenerates into a TS. We will discuss FCNs that possess only one single-core processor or just utilize a single-core for fog computing like a great number of routers and switches which support fog computing. 

Some papers have modeled the FCN as an M/M/1 queuing system [3,4,5] that uses an FCFS mechanism to arrange tasks in the task deque, where they wait until the processor idle. However, since the M/M/1 queuing system is no longer appropriate for fog computing, this paper proposes a TS system perfectly suited to fog computing. Furthermore, this paper puts forward an important measure of FCN performance—the concurrency coefficient—which denotes the expected response time for one task with a specific number of instructions. The concurrency coefficient is an important measurement for multi-class tasks. In [6,7], the authors merely analyze single-class tasks, which is unrealistic for various IoT applications.

In fog computing, heterogeneous FCNs differ in processing rate and input load, the heterogeneity gives rise to a load imbalance. For example, some weak FCNs become over-loaded while some strong FCNs remain idle. The imbalance largely decreases the processing capacity of the whole fog computing network and increases the response time for IoT applications. Accordingly, we propose a load-balancing algorithm that guides FCNs to collaborate by an adjusted work-stealing scheduler, which is decentered in contrast to its counterpart. Moreover we prove that the scheduling algorithm can achieve Pareto optimality based on the Nash bargaining solution.

At the end of this paper, some simulations are summarized. The simulation results prove that our load balancing algorithm can reduce the response time more than the classical work-stealing algorithm, especially for light tasks.

This paper is organized as follows:

- Section 2 introduces related progress on collaborative load-balancing algorithms for IoT;
- Section 3 analyzes the FCN working mechanism, proposes a work-stealing algorithm for a TS system and solves the probability allocation problem by means of the Nash bargaining solution;
- Section 4 elaborates the simulation results and proves the validity and efficiency of GWS; and
- Section 5 concludes the content and proposes future work.

## 2. Related Work

In fog computing, FCNs are always modeled as an M/M/1 queuing system [3,4,5], which adopts the first-come-first-serve (FCFS) mechanism, however, this mechanism no longer satisfies the needs of IOT applications, especially for wireless sensor and actuator networks which require a quick response. Section 3.1 proves that the FCFS mechanism leads to a fixed delay for any tasks. It is poor for applications which are full of light tasks, like wireless sensor and actuator networks. Light tasks are those need little processing time and need a response as quickly as possible.

Load balancing algorithms fall into two categories: work-sharing and work-stealing, or static and dynamic. ‘Work-sharing’ means that all tasks are dispatched to other processors through a center, whereas ‘work-stealing’ means that tasks are stolen from processors proactively. ‘Static’ means that the algorithm rules are predefined, and ‘dynamic’ means that the algorithm rules are decided in run time and always changing. For FCNs, the dynamic work-stealing algorithm is best, because a fog computing network is versatile with tasks arriving continually. A classical work-stealing scheduler was proposed in [8], which applies to multiprocessors for multithreaded applications. In a fog computing network, an FCN can also be viewed as a processor; however, there are still some vital differences between an FCN and a processor. For example, the memory swap and synchronization between processors is much faster and easier than in FCNs, which are located at different sites. 

Moreover, the work-stealing for TS systems is rather difficult and expensive to implement practically [9]. Thus, Section 3.2 proposes GWS to steal raw tasks from the residual queue. Moreover, [7] explores how to share tasks in a TS system; however there is only one task input source, and all tasks must be allocated through it. This is unrealistic for fog computing, where there are numerous dispersed task input sources.

Another work-stealing strategy was proposed by routing the stealing and response based on the trajectories of moving users [10]. The author predicts future users’ addresses according to their historical trajectory; however the trajectory prediction may be imprecise, and a large number of users would cause too much overhead to calculate trajectories and manage routes. In fog computing, the number of different users and applications is too large for a prediction algorithm. So we insist that the work-stealing strategy be succinct to avoid heavy computing load and network congestion.

A load-balancing algorithm for single-class tasks was proposed in [6], which treated the load balancing problem as a cooperative game between processors based on game theory. We propose a scheduling algorithm for multi-class tasks.

Another hierarchical work-stealing algorithm was proposed for reducing the stealing frequency [11] by clustering FCNs and selecting one FCN as the leader; however central management may give rise to a single point of failure, as FCNs are not very stable. This paper avoids selecting one FCN as the leader and instead takes full advantage of the cloud, which is powerful and stable. With the help of the cloud, we can efficiently manage load balancing between FCNs.

Considering the modern trend of Big Data, an overview for Big IoT Data Analytics was proposed by [12], and a survey of service migration in edge computing was conducted by [13]. These all are promising aspects of fog computing in IoT applications.

## 3. Collaborative Work-Stealing Algorithm

### 3.1. FCN Working Mechanism

**Figure 2:**
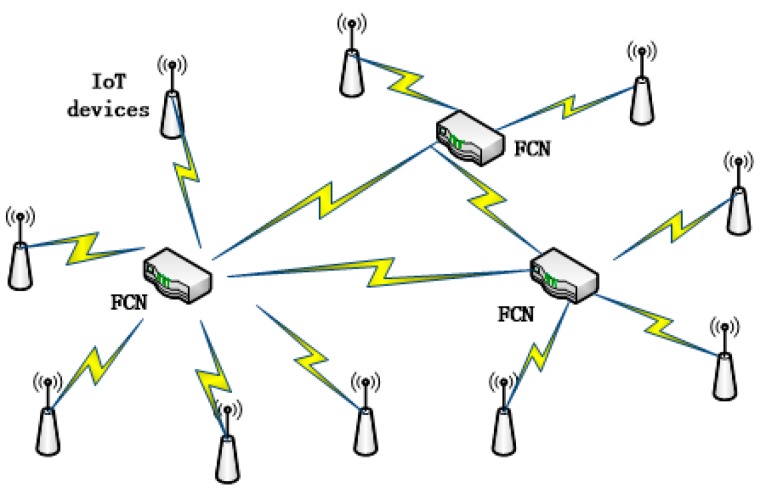
Fog computing nodes and IoT devices nearby.

Fog computing is a virtual service for IoT applications. FCNs can be routers, switchers and other network devices that support fog computing service. FCNs serve other IoT devices nearby by helping to process the tasks that arrive from them as Figure 2 shows.

**Figure 3:**
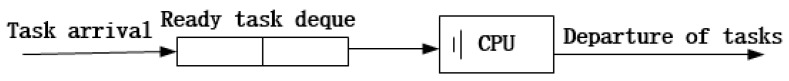
Fog computing node of M/G/1 model.

Since the tasks’ sources are various and arrival intervals are random, the arrival process can be modeled as a Possion process. We assume the arrival rate as λ. For task processing, we do not assume the service time as an exponential distribution as [3,4,5], as that is not reasonable. We discuss the general distribution below. The system is shown in Figure 3. The task is stored in one deque, which is a variety of queues that permit in-out operations from both ends [14].

Thus the FCN is a M/G/1 queuing system with the following properties: the arrival rate is λ, the process time is $T_{service}$, $E\left(T_{service}\right)$ denotes the average service time per task, and $E\left({T_{service}}^{2}\right)$ denotes the second moment of $T_{service}$. According to [15], the M/G/1 queuing system holds the following two equations:

(1) $$T_{response}=T_{wait}+T_{service},$$

(2) $$\rho =\lambda E\left(T_{service}\right),$$

(3) $$E\left(T_{wait}\right)=\frac{\lambda E\left({T_{service}}^{2}\right)}{2\left(1-\rho \right)},$$

In the above equations ρ denotes load intensity, $T_{wait}$ denotes the waiting time in the queue and $T_{response}$ denotes response time which equals a task’s entire time in the FCN. As the FCN abides by the FCFS mechanism, arriving tasks first wait in the ready queue then receive service until the processor is idle, so the $T_{response}$ consists of $T_{wait}$ and $T_{service}$. Here we propose a new important measure factor $E(T_{response}|T_{service}=x)$ which denotes the conditional expectation of $T_{response}$ while $T_{service}$ is set to x. Since how long one task waits is independent of its service time, we obtain the following equation:

(4) $$E\left(T_{response}|T_{service}=x\right)=E\left(T_{wait}\right)+x.$$

Equations (3) and (4) can be simplified as:

(5) $$E\left(T_{response}|T_{service}=x\right)=\frac{\lambda E\left({T_{service}}^{2}\right)}{2\left(1-\rho \right)}+x.$$

**Figure 4:**
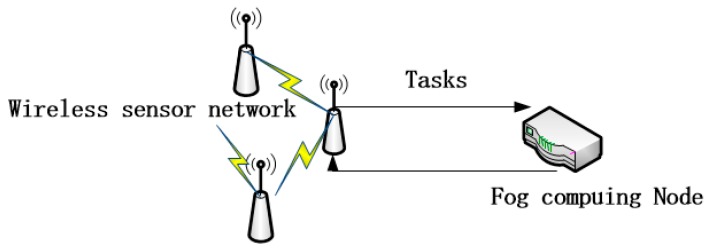
Wireless sensor network applications.

According to Equation (5), we find that no matter how small a task is, the response time can be no shorter than $\frac{\lambda E\left(T_{service}{}^{2}\right)}{2\left(1-\rho \right)}$, which is the lower bound limit of time; however, some sense-and-actuate-loop IoT applications like a wireless sensor and actuator network, which generate a large number of light tasks and demand quick response time, will obtain terrible QoS due to the unavoidable lower-bound time limit, so the FCFS system is not appropriate for the FCN. We propose a classical mechanism that can cut off the lower-bound time limit–time-sharing (TS) mechanism. A TS mechanism is efficient for concurrency, which has been applied to computer systems successfully. As Figure 4 shows, the wireless sensor shunts its tasks to the FCN for service, and the time-sharing FCN can respond quickly and provide high QoS.

**Figure 5:**
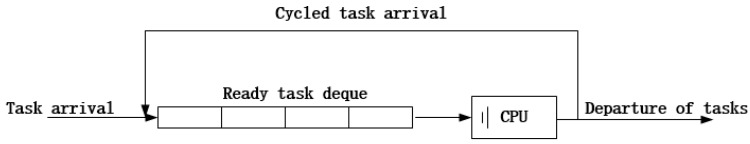
Time-sharing system model.

Next, we elaborate the TS system. As Figure 5 shows, once a task arrives, it is pushed into the task deque from the back. The CPU obtains a task from the front of the task deque and gives it a little quantum of service. When the task is completed, it leaves the FCN; otherwise, it is pushed into the task deque from the back, which is called ‘cycled arrival’. Through time-sharing, each task receives service in turn. The service quantum is so little that every task seems to be served at the same time. The scheduling mechanism is also called Round-Robin (RR) time-sharing. According to [16], we obtain a key equation:

(6) $$E\left(T_{response}|T_{service}=x\right)=\frac{x}{1-\rho }.$$

so if FCN adopts the RR algorithm, the expectation of $T_{stay}$ is proportional to $T_{service}$. Some IoT applications, like wireless sensor and actuator networks which are comprised of small tasks, can obtain a quick response in contrast to waiting for a fixed time in the FCFS system. in Section 4, we compare the two scheduling algorithms through simulations.

We compare Equations (5) and (6), and we find that although the TS system removes the lower- bound time limit, its coefficient for service time is larger than that of FCFS system: $\frac{1}{1-\rho }>1$. So the TS system decreases the response time for light tasks at the expense of increasing the response time for heavy tasks. But to be reasonable, the light tasks always demand more than heavy tasks. So we believe that the expense is reasonable.

We also found that the TS system has a potential advantage, as Equation (7) shows:

(7) $$\frac{E\left(T_{response}|T_{service}=x\right)}{x}=\frac{1}{1-\rho }.$$

The ratio between the expected $T_{response}$ and $T_{service}$ is constant, which just relates to ρ; however ρ is decided by λ and $E\left(T_{service}\right)$, so the TS scheduler is completely fair to all tasks, whether small or large. This feature is necessary and helpful. As if the ratio is smaller for small tasks, users and developers tend to split a large task into smaller tasks. Or if the ratio is larger for small tasks, then users and developers tend to merge small tasks into larger ones for a quicker response. Such unfairness will cause malicious competition and add the burden of users and developers. So we insist that fairness is necessary in fog computing, which means that $\frac{E\left(T_{response}|T_{service}=x\right)}{x}$ is the same for any task processed in any FCN.

In a FCN, one task may gain different $T_{service}$ in different FCNs. The service time of one task depends on its programming architecture and the processors of the serving FCN. For convenience we propose on absolute value π that denotes the number of instructions of one task to represent the working load of tasks and an absolute value s that denotes the number of instructions processed by the FCN per unit time. We obtained modified equations as follows:

(8) $$E\left(T_{service}\right)=\frac{E\left(\pi \right)}{s},$$

(9) $$\mu =\frac{1}{E\left(T_{service}\right)}=\frac{v}{E\left(\pi \right)},$$

(10) $$\rho =\frac{\lambda }{\mu }=\frac{\lambda *E\left(\pi \right)}{s},$$

(11) E(Tresponse|π=x)x=E(Tstay |Tservice=xv)xs∗ 1s=1s∗(1−ρ),

(12) $$C=\frac{E\left(T_{response}|\pi =x\right)}{x}=\frac{1}{s*\left(1-\rho \right)}=\frac{1}{s-\lambda *E\left(\pi \right)},$$

Here, C denotes the FCN’s performance which is called the ‘concurrency coefficient’. As we can see, the smaller C means quicker response and better performance of FCNs. So we aim to reduce C. And C is dependent on the processing rate of the FCN, the arrival rate, and the average service time of tasks. The next section will elaborate a new scheduler that aims to minimize concurrency coefficients of FCNs based on game theory. 

### 3.2. Game-Theory Based Work-Stealing Scheduler

Work-stealing as a scheduling algorithm is imposed on a multi-processor system to balance the load between processors. Compared to a multi-processor system, a fog computing network features a much more complex topology, more significant communication delay and dispersive memory. Stealing between FCNs is much more expensive than between processors. A normal work-stealing scheduler adopts a strategy where an idle processor steals from a randomly chosen processor. If the victim processor has more than one task, it transfers the extra task to the stealer; otherwise, it responds with a refusal command and then the stealer attempts another randomly chosen processor. In conclusion, if the scheduler is applied to a fog computing network, it suffers from the following defects:

1. Idle FCNs must wait until they successfully steal a task, which wastes time and energy.
2. A TS system is very hard and costly for the dispersive memory distribution [9]. So we ought to adjust the normal work-stealing algorithm for FCNS, which adopts the TS mechanism.

We have to adjust the normal work-stealing scheduler for fog computing. Fog computing is the complement to cloud computing (as Figure 1 shows), as every FCN is connected to the cloud. So we can utilize the cloud to help with work-stealing. A cloud manages a cluster of FCNs and orchestrates their cooperation. The parameters of the cluster of FCNs are listed in Table 1.

In the above list, some parameters are almost fixed, like $s_{i}$, which denotes the processing capacity of the FCN. The other parameters should be bookkept by the FCN itself and reported to the cloud periodically.

In the fog computing network above, every FCN has its own concurrency coefficient based on Equation (12). This factor is an important measure of performance which denotes the average response time of the specified task. For $F_{i}$ the concurrency coefficient is in Equation (13):

(13) $$C_{i}=\frac{1}{s_{i}-\lambda_{i}*\bar{\pi_{i}}},\;i=1,2,\ldots ,N.$$

Obviously, a different FCN may possess a different processing rate $\partial_{i}$, different task arrival rate $\lambda_{i}$ and different average instruction number per task $\bar{\pi_{i}}$, which may lead to different concurrency coefficient $C_{i}$ based on the Equation (13). The goal of this paper is to fairly achieve a larger $C_{i}$ for every FCN.

From the Equation (13), for an FCN like $F_{i}$, the concurrency coefficient $C_{i}$ only depends on $s_{i}$, $\lambda_{i}$ and $\bar{\pi_{i}}$. Among these factors, $s_{i}$, which denotes the process rate of $F_{i}$, is almost fixed; $\lambda_{i}$, which denotes the task arrival rate of $F_{i}$, just relies upon the IoT devices in this area; and $\bar{\pi_{i}}$ denotes the average number of instructions per task of $F_{i}$. So the only factor that can be modified is the task arrival rate $\lambda_{i}$. We can modify $\lambda_{i}$ so that $C_{i}$ approximates the average value of the concurrency coefficient $\bar{C}$. The algorithm is elaborated as follows.

**Figure 6:**
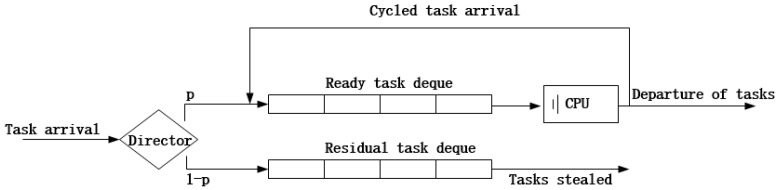
Over-loaded FCN model (*p* < 1).

**Figure 7:**
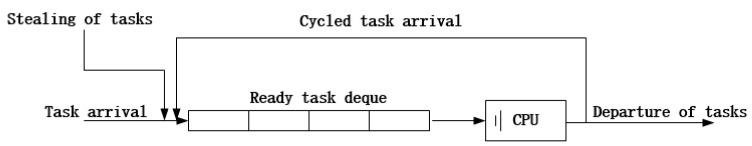
Under-loaded FCN model (*p* > 1).

This adjusted work-stealing algorithm aims to adjust the task input intensity. We classify FCNs into two varieties: over-loaded FCNs with a large concurrency coefficient and under-loaded FCNs with a small concurrency coefficient. The work-stealing algorithm reduces the task arrival rate of over-loaded FCNs and raises the task arrival of under-loaded FCNs by shunting and stealing. The two varieties of FCNs are modeled in Figure 6 and Figure 7.

As Figure 6 shows, an FCN contains two task deques, the ready task deque, which works with the CPU, and the residual task deque, which stores raw tasks that are ready to be stolen. Another big difference is the director, which decides whether a task goes to the ready or residual task deque on the probability of p. So what is *p*? This will be discussed in Section 3.3. For over-loaded FCNs like $F_{i}$, all of the arriving tasks go to the ready task deque at the probability of $p_{i}$, so the task arrival rate becomes $\lambda_{i}*p_{i}$. The updated concurrency coefficient is expressed as follows:

(14) $$C_{i}^{\prime }=\frac{1}{s_{i}-\lambda_{i}*p_{i*}\;\bar{\pi_{i}}}.$$

When $p_{i}$ is less than 1, the director of this FCN will shunt arriving tasks to the ready task deque at the probability of $p_{i}$ and to the residual task deque at the probability of $1-p_{i}$. Tasks in the ready task deque will receive service one by one, and tasks in the residual task deque wait for a stealing request. Once a task enters the ready deque, it cannot be shared; tasks in in the residual dequee are raw and suitable for sharing. When a stealing request comes, and the residual task deque is not empty, the FCN delivers a task from the back of the residual task deque to the stealing FCN.

When $p_{i}$ is greater than 1, as shown in Figure 7, the FCN is under-loaded. There is no need to maintain the residual deque as the director. The FCN has to steal another FCN so that the overall task arrival rate can increase. We set the successful stealing interval as an exponential distribution with the average value of $\lambda_{i}*\left(p_{i}-1\right)$. A successful stealing interval means the interval between two stealing from FCS which contains extra tasks. If the FCN fails to steal, it continues without waiting.

An exceptional situation occurs when $p_{i}$ is equal to 1, then FCN is just the same as an isolated TS system that does not steal or shunt any tasks to the residual task deque. This special case rarely happens, so we don’t discuss this in the following sections.

As the cloud periodically updates the FCN cluster, the role of each FCN may change. Some over-loaded FCNs may become under-loaded and vice versa, so the algorithm is dynamic to the real IoT environment and evolves periodically. In next section, we will discuss how to calculate the probability set $p=\left\{p_{1},p_{2},\ldots ,p_{N}\right\}$. This is the key factor for our algorithm.

### 3.3. Nash Bargaining Solution for the Probability Set

**Figure 8:**
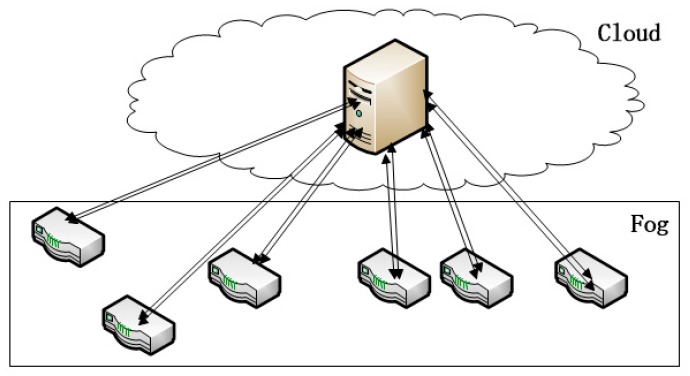
Cooperative game between FCNs.

Section 3.2 proposes an efficient scheduling algorithm of work-stealing for a TS system, but how to set the important factor $p_{i}$ has not be solved. The main goal of the paper is to minimize the concurrency coefficient $C_{i}^{\prime }\left(p_{i}\right)$ of each FCN. The problem can be modeled as a NBS rather than a Nash equilibrium for cooperative FCNs. This is a cooperative game, which is different from its non-cooperative counterpart [17]. Through cooperation of players (FCNs), a better profit can be achieved. The game is depicted in Figure 8.

As Figure 8 shows, FCNs gain common knowledge through the cloud. FCNs can communicate with each other to gain common knowledge, but this communication process is of time complexity O(N∗N). So why not draw support from the cloud? As all FCNs are linked to the cloud, so FCNs can gain all information needed for the game by means of the central cloud with the time complexity O(N). Although the cloud may be far from edge, I believe a decrease in time complexity by one order of magnitude can offset it even more. Let’s analyze the game to find the optimal balancing points. The mathematical problem is as follows:

(15) $$\min C_{i}^{\prime }\left(p_{i}\right),\;i=1,2,\ldots ,N,$$

(16) $$p_{i}>0,$$

(17) $$p_{i}<\frac{s_{i}}{\lambda_{i}*\;\bar{\pi_{i}}},$$

(18) $$\overset{N}{\underset{i=1}{\sum }}\lambda_{i}*p_{i}=\lambda ,$$

Inequation (16) guarantees the probability $p_{i}$ is not negative and Inequation (17) is the stability condition of the M/G/1 queuing system. We replace $C_{i}\left(p_{i}\right)$ according to Equation (14), so objective (14) can be simplified as:

(19) $$\max\;\left(\Phi p_{i}\right)=-p_{i},\;i=1,2,\ldots ,N.$$

By maximizing $\Phi (p_{i})$, $F_{i}$ minimizes $C_{i}\left(p_{i}\right)$ at the same time. Every FCN cooperates by means of the cloud center to gain better performance. This problem can be viewed as a Nash bargaining game for cooperative players. According to [18], the NBS can realize Pareto optimal operation point; that is, NBS guarantees optimality and fairness for every FCN. According to [17], the above objective (19) is equivalent to the following objective:

(20) $$\max\overset{N}{\underset{i=1}{\prod }}\left(\Phi \left(p_{i}\right)-\eta_{i}^{0}\right),$$

where $\eta_{i}^{0}$ indicates the initial agreement point which denotes that $\Phi (p_{i})$ must not be less than $\eta_{i}^{0}$. We set $\eta_{i}^{0}=-\frac{s_{i}}{\lambda_{i}*\;\bar{\pi_{i}}}$ based on Inequation (17) and we set

(21) $$\psi_{i}=-\eta_{i}^{0}=\frac{s_{i}}{\lambda_{i}*\;\bar{\pi_{i}}}$$

In conclusion, the above problem can be elaborated as follows:

(22) $$\max\prod_{i=1}^{N}\left(\Phi \left(p_{i}\right)-\eta_{i}^{0}\right)=\prod_{i=1}^{N}\left(\psi_{i}-p_{i}\right),p_{i}>0.$$

Then we use the Lagrange multiplier method to find the set $p=\left\{p_{1},p_{2},\ldots ,p_{N}\right\}$ for the maximum objective. But first we ignore the condition $p_{i}>0$ and apply it later. The Lagrange function is as follows, and u and $v_{i}$ are multipliers for Equation (18) and Inequation (17).

(23) $$L\left(p_{i},u,v_{i}\right)=\overset{N}{\underset{i=1}{\sum }}In(\psi_{i}-p_{i})+u*\left(\overset{N}{\underset{i=1}{\sum }}p_{i}*\lambda_{i}-\lambda \right)+\overset{N}{\underset{i=1}{\sum }}v_{i}*\left(p_{i}-\psi_{i}\right).$$

Then we apply the Karush Kuhn Tucker (KKT) constraints as follows:

(24) $$\frac{\partial L}{\partial p_{i}}=\frac{1}{p_{i}-\psi_{i}}+\lambda_{i}*u+v_{i},$$

(25) $$\frac{\partial L}{\partial u}=\overset{N}{\underset{i=1}{\sum }}p_{i}*\lambda_{i}-\lambda =0,$$

(26) $$v_{i}*\left(p_{i}-\psi_{i}\right)=0.$$

According to (17) and (19), we know $p_{i}<\psi_{i}$, so we deduce $v_{i}=0$, and Equations (24)–(26) can be concluded as:

(27) $$\frac{1}{p_{i}-\psi_{i}}+\lambda_{i}*u=0,$$

(28) $$\overset{N}{\underset{i=1}{\sum }}p_{i}*\lambda_{i}-\lambda =0,$$

The result set $p=\left\{p_{1},p_{2},\ldots ,p_{N}\right\}$ can be resolved from Equations (25) and (26).

(29) $$p_{i}=\psi_{i}-\frac{\sum_{i=1}^{N}\lambda_{i}*\psi_{i}-\lambda }{\lambda_{i}*N}.$$

Until now the result set $p=\{p_{1},p_{2},\ldots ,p_{N}$} has not been solved because the constraint (16) $p_{i}>0$ has not been applied. If $p_{i}<0$, Equation (16) infers that $\psi_{i}$ is too little. But based on (21), if $\psi_{i}$ is too small, then weak processing capacity is too weak and task arrival too frequent—both of which lead to FCN failure. So we just abandon FCNs with negative $p_{i}.$ According to [7], we can remove the $F_{i}$ for which $p_{i}<0$ by using the following algorithm in the time complexity of O(n∗log(n)).

In the above algorithm, the sorting accounts for the time complexity of O(n∗log(n)). The Pareto optimal point is calculated out as $p=\{p_{1},p_{2},\ldots ,p_{N}$}. In the fog computing network, we adopt the result set $p=\{p_{1},p_{2},\ldots ,p_{N}$} to implement the work-stealing scheduler, and the Pareto optimal maximum for the concurrency coefficient will be achieved. ‘Pareto optimality’ means there is no way to improve performance of one FCN without decreasing the performance of others.

The next section elaborates the simulations that prove the efficiency of the Algorithm 1 below.
**Algorithm 1:** post-processing algorithm for eliminating the negative $p_{i}$**Input**: task arrival rate $\lambda_{i}$, the overall task arrival rate *λ*, the parameter $\psi_{i}$ and the FCN number N.
**Output**: probability set $p=\left\{p_{1},\;p_{2},\ldots ,\;p_{N}\right\}$.
Sort all FCNs in decreasing order of $\psi_{i}$,$\Phi =\frac{\sum_{i=1}^{N}\lambda_{i}*\psi_{i}-\lambda }{N},$**While**$\left(\psi_{i}<\frac{\Phi }{\lambda_{i}}\right)$$p_{i}=0$n=n−1,$\Phi =\left(\Phi -\frac{\lambda_{n+1}*\psi_{n+1}}{n+1}\right)*\frac{n+1}{n}$**end while****for**i=1,2,…,n$p_{i}=\psi_{i}-\frac{\Phi }{\lambda_{i}},$**end for.**


## 4. Simulations

Lastly some simulations were completed to prove the efficiency of GWS. The simulations were programmed in C++ language, and the figures were drawn using OriginPro 2016. 

Simulation I is a comparison between the FCFS mechanism and TS mechanism on an FCN. In [3,4,5], the FCN adopts the FCFS mechanism, and we suggest the TS mechanism, which was proved in Section 2. We perform a simulation where one FCN adopts the FCFS mechanism while another adopts the time-sharing, and other parameters like task input and processing rate are kept equal. The specific parameters are as in Table 2.

The four nodes possess the same task input and just differ in work mechanism and processing rate of 200 M and 400 M. The 200 M and 400 M here mean the CPU dominant frequency. A 200 M CPU can perform work of 200M clock periods per second. As an instruction needs 8 clock periods, so this CPU can complete 25 M instructions per second, and a 400 M CPU can complete 50 M instructions per second.

**Figure 9:**
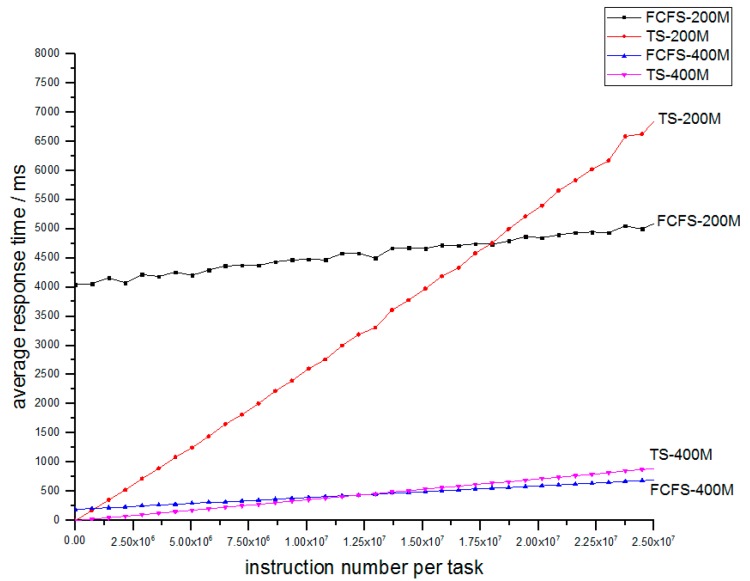
Relation of average response time and number of instructions for FCFS-200M and TS-200M, FCFS-400M and TS-400M.

We can obtain the relation between service time and stay time as shown in Figure 9. The FCFS system contains a lower-bound limit of response time around 4000 ms, which is not suitable for some IoT applications especially wireless sensor networks. By contrast, TS is just appropriate for wireless sensor network applications because it avoids the lower bound. So for light tasks, the TS mechanism can guarantee very good performance. But when tasks are heavy, the TS mechanism needs more response time, in contrast to FCFS system. This is just the expense of a TS mechanism, as Section 3.1 proves. By comparing nodes of different process rates, we can find that a higher processing rate means a shorter response time. Meanwhile, when the processing rate increases, the line of FCFS and TS become closer. The reason is that when process rate increase, the work strength decreases, which means that the work deque always only contains one or fewer tasks. Then the TS and FCFS mechanism are the same.

**Figure 10:**
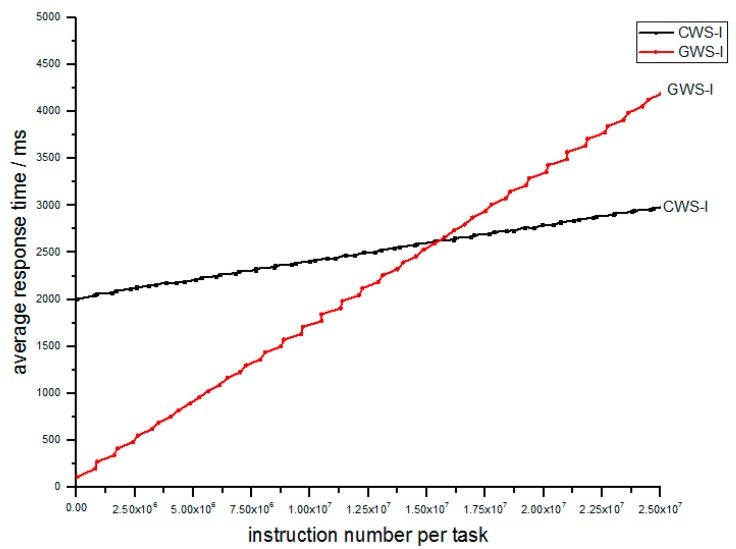
Relation of average response time and number of instructions per task between CWS-I and GWS-I.

Simulation II focuses on the performance of the whole cluster of 100 FCNs. The parameters are shown in Table 3; $\bar{\lambda }$, $\bar{s}$, $\bar{\pi }$ and N separately denotes the average task arrival rate, the average processing rate, the average number of instructions per task and the number of FCNs in the cluster of FCNs. A fog computing network, which separately adopts CWS or GWS receives the same task input over a long period. Let’s see the performance according to the relation between the number of instructions and the response time in Figure 10. We find that GWS outperforms CWS. By means of GWS, the IoT task can achieve much faster response, especially for light tasks, but as for heavy tasks, the GWS needs more time. It is worthwhile because heavy tasks always hold loose time limits compared to light ones.

Then we explore how the working load influences the fog computing network by changing the work load of the whole system. Three simulations will be carried out to explore the influence of average arrival rate, average processing rate and average number of instructions per task.

**Figure 11:**
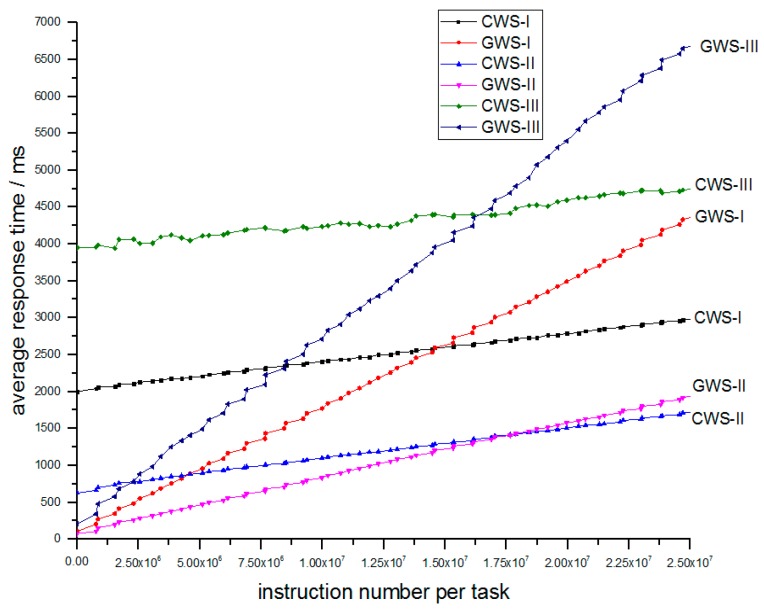
Relation of average response time and number of instructions per task between different arrival rates.

Simulation III explores the influence of arrival rate. In Table 4, we just change the average arrival rate of tasks and other parameters maintain the same. The experiment result of simulation III is depicted in Figure 11.

As we can see from Figure 11, no matter how arrival rate changes, the corresponding relation of CWS and GWS never changes. When arrival rate go bigger, the lines of CWS rise and the lines of GWS steepen. The CWS-III obtains bigger time lower bound than CWS-I and CWS-II, meanwhile GWS-III obtains bigger response time than GWS-I and GWS-II. But the GWS ones can still maintain less response time compared to CWS ones for light tasks.

Simulation IV explores how average processing rate influences performance. The parameters are shown in Table 5.

**Figure 12:**
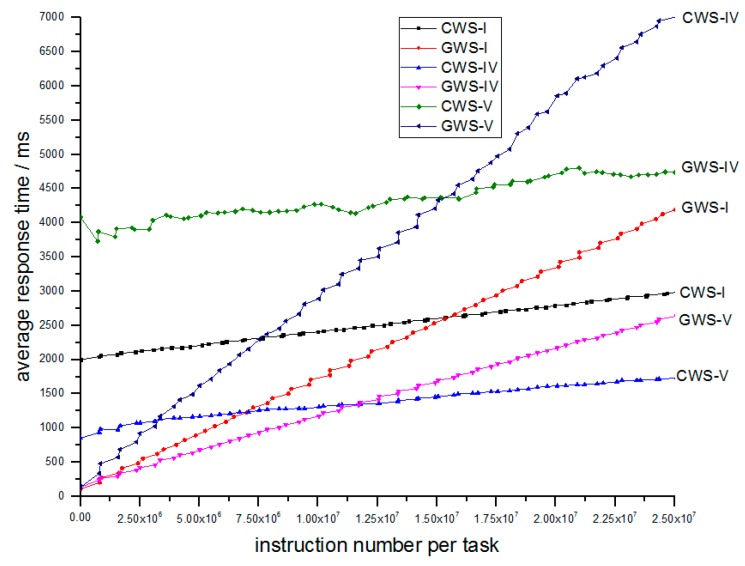
Relation of average response time and number of instructions per task between different process rates.

As we can see from Table 5 that only processing rate is different. The experimental result is depicted in Figure 12.

In the Figure 12, the relative relation of CWS-IV and GWS-IV, or CWS-V and GWS-V remains as CWS-I and GWS-I, because GWS-IV and GWS-V still obtain less response time than their counterparts for light tasks. And while process rate increases, the response time also decreases.

Simulation V studies the influence of average number of instructions per task. The related parameters are shown in Table 6.

**Figure 13:**
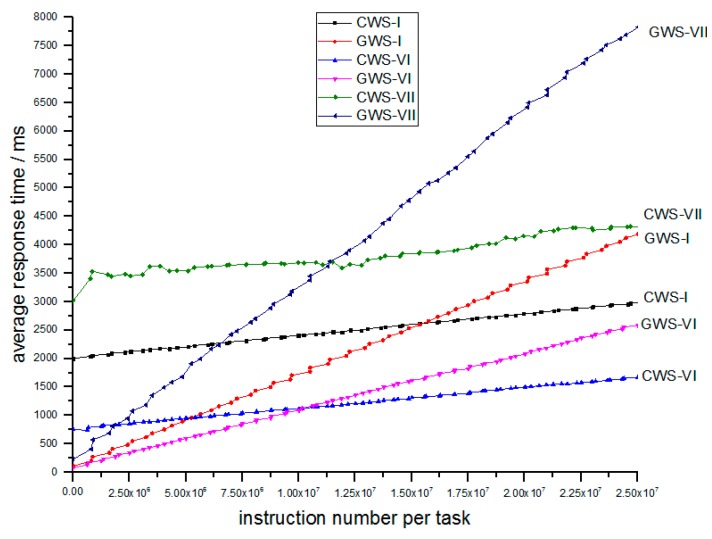
The relation of average response time and number of instructions per task between different average instruction number per task.

In Table 6, the networks only differ in average instruction number. The experimental result is shown in Figure 13.

In Figure 13 above, we can find that the GWS-VI and GWS-VII are still better for light tasks, and while average number of instructions per task increases, the response time also increases.

**Figure 14:**
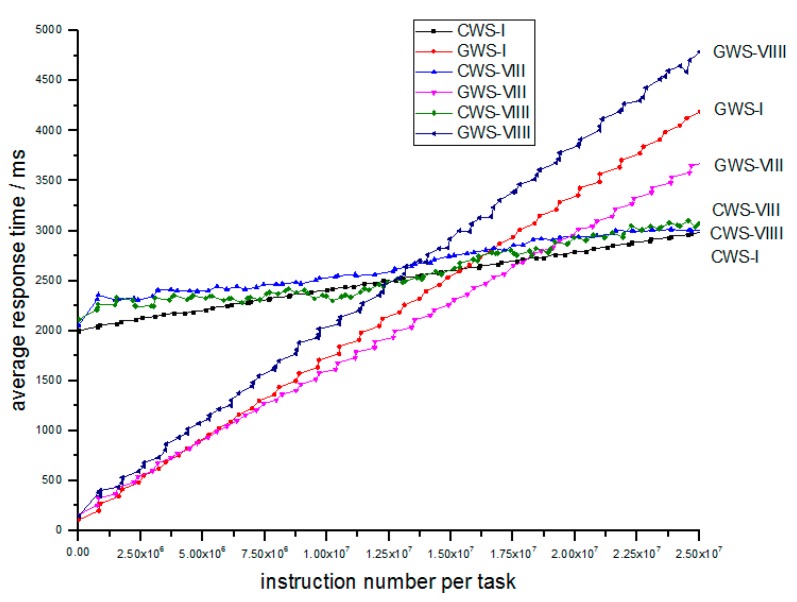
Relation of average response time and number of instructions per task between different FCN number.

Finally in Simulation IV, we explore the scalability of GWS by changing the FCN number N from 100 to 20 and 100 to 500. Parameters are shown in Table 7. The results are depicted in Figure 14.

According to Figure 14, we find that the GWS is well scalable for network size as the slopes of the two lines are almost the same, so when the FCN cluster grows bigger, the GWS is still stable and robust which is necessary for fog computing. This feature is proved in NVS which is size-irrelevant.

From the simulations above, we conclude that by increasing average task arrival rate, decreasing average process rate and increasing average number of instructions per task, the response time of CWS and GWS will increase but the relative relation never changes. The GWS network always gives better performance for light tasks than the CWS network and the GWS is scalable as it is size-irrelevant.

## 5. Conclusions

Firstly, this paper clarifies the task processing mechanism of FCNs and proposes to replace the FCFS mechanism with the TS mechanism in FCNs. Then the validity and necessity of the TS mechanism is proved in both theory and simulation. A measurement of FCN performance for multi-class tasks is also put forward as the concurrency coefficient.

Secondly, the paper adjusts the work-stealing algorithm for the TS system by setting up residual deques for raw tasks and stealing raw tasks from other residual deques. A variant of the work-stealing algorithm is modeled as a cooperative game between FCNs.

Finally, the paper proposes a collaborative algorithm GWS to balance the load between FCNs. The collaborative algorithm is a variant of work-stealing, which is based on game theory. According to the Nash bargaining solution, we can obtain Pareto optimality. Through simulations, we prove that GWS can obtain better performance than CWS scheduler, especially for light tasks.

The paper introduces GWS, which places FCNs in collaboration with each other to achieve better performance; however, cooperation between FCNs like task-stealing causes an information swap, so our future studies will examine privacy security for IoT applications. We will aim to study safe ways of cooperating without leaking any user information.

## Figures and Tables

**Table 1:** Parameter names and paraphrases.

Parameter Name	Parameter Paraphrase
N	Number of FCNs in this cluster
$F_{i}$	The $i_{th}$ FCN
$\lambda_{i}$	Average task arrival rate of $F_{i}$
$\bar{\pi_{i}}$	Average instruction number per task of $F_{i}$
$s_{i}$	Processed instruction number per unit time of $F_{i}$
$\mu_{i}$	Number of processed tasks per unit time of $F_{i}$
$C_{i}$	Concurrency coefficient of $F_{i}$

**Table 2:** Parameters of Simulation I.

FCN Name	*λ*	s	$\bar{\pi }$
FCFS-200M	1.2	2.5 × 10^7^	1 × 10^7^
TS-200M	1.2	2.5 × 10^7^	1 × 10^7^
FCFS-400M	1.2	5 × 10^7^	1 × 10^7^
TS-400M	1.2	5 × 10^7^	1 × 10^7^

**Table 3:** Parameters of Simulation II.

Network Name	$\bar{\lambda }$	$\bar{s}$	$\bar{\pi }$	N
CWS-I	1.2	2.5 × 10^7^	1 × 10^7^	100
GWS-I	1.2	2.5 × 10^7^	1 × 10^7^	100

**Table 4:** Parameters of Simulation III.

Network Name	$\bar{\lambda }$	$\bar{s}$	$\bar{\pi }$	N
CWS-I	1.2	2.5 × 10^7^	1 × 10^7^	100
GWS-I	1.2	2.5 × 10^7^	1 × 10^7^	100
CWS-II	0.8	2.5 × 10^7^	1 × 10^7^	100
GWS-II	0.8	2.5 × 10^7^	1 × 10^7^	100
CWS-III	1.4	2.5 × 10^7^	1 × 10^7^	100
GWS-III	1.4	2.5 × 10^7^	1 × 10^7^	100

**Table 5:** Parameters of Simulation IV.

Network Name	$\bar{\lambda }$	$\bar{s}$	$\bar{\pi }$	N
CWS-I	1.2	2.5 × 10^7^	1 × 10^7^	100
GWS-I	1.2	2.5 × 10^7^	1 × 10^7^	100
CWS-IV	1.2	2 × 10^7^	1 × 10^7^	100
GWS-IV	1.2	2 × 10^7^	1 × 10^7^	100
CWS-V	1.2	3 × 10^7^	1 × 10^7^	100
GWS-V	1.2	3 × 10^7^	1 × 10^7^	100

**Table 6:** Parameters of Simulation V.

Network Name	$\bar{\lambda }$	$\bar{s}$	$\bar{\pi }$	N
CWS-I	1.2	2.5 × 10^7^	1 × 10^7^	100
GWS-I	1.2	2.5 × 10^7^	1 × 10^7^	100
CWS-VI	1.2	2.5 × 10^7^	0.8 × 10^7^	100
GWS-VI	1.2	2.5 × 10^7^	0.8 × 10^7^	100
CWS-VII	1.2	2.5 × 10^7^	1.2 × 10^7^	100
GWS-VII	1.2	2.5 × 10^7^	1.2 × 10^7^	100

**Table 7:** Parameters of Simulation VI.

Network Name	$\bar{\lambda }$	$\bar{s}$	$\bar{\pi }$	N
CWS-I	1.2	2.5 × 10^7^	1 × 10^7^	100
GWS-I	1.2	2.5 × 10^7^	1 × 10^7^	100
CWS-VIII	1.2	2.5 × 10^7^	1 × 10^7^	20
GWS-VIII	1.2	2.5 × 10^7^	1 × 10^7^	20
CWS-VIIII	1.2	2.5 × 10^7^	1 × 10^7^	500
GWS-VIIII	1.2	2.5 × 10^7^	1 × 10^7^	500
